# Case Report: Scleral hyperpigmentation associated with oral hydroxychloroquine use

**DOI:** 10.3389/fopht.2023.1192019

**Published:** 2023-06-05

**Authors:** Caroline Maretz, Felix Omoruyi, Rachel A. F. Wozniak

**Affiliations:** Department of Ophthalmology, University of Rochester School of Medicine and Dentistry, Rochester, NY, United States

**Keywords:** sclera, hyperpigmentation, drug reaction, hydroxychloroquine, skin pigmentation

## Abstract

Hydroxychloroquine (HCQ) is a commonly used medication for its immunosuppressive and dermatologic effects. It has known ocular side effects, which include retinopathy, corneal deposits, and choroidal thinning. Herein, we report the first known case of HCQ-induced hyperpigmentation of the sclera. A 75-year-old female presented after 10 months of gradual progression of painless blue-gray discoloration of the bilateral sclera, fingernails, and lower extremities secondary to oral HCQ therapy. Cessation of the drug led to a partial reversal of the hyperpigmentation at 5 months, further supporting HCQ as the causative agent. Hyperpigmentation reactions can be distressing to patients and lead to decreased medication adherence; given the widespread use of HCQ, it is important to increase awareness of this potential drug reaction.

## Introduction

Ocular hyperpigmentation is defined as an acquired, painless condition in which patches of an oculocutaneous tissue darken relative to the surrounding tissue. These reactions commonly co-occur in the sclera and mucocutaneous tissues, and they can be cosmetically undesirable for the patient. Scleral hyperpigmentation may be associated with underlying ocular and systemic diseases including scleromalacia perforans, Addison’s disease, ochronosis, and melanoma, and drug-induced causes. It is important to identify cases of drug-induced hyperpigmentation, as it can affect adherence to treatment (1). Furthermore, signs of discoloration can, in some cases, resolve with cessation of the causative drug (2).

There are a wide range of systemic medications associated with ocular or cutaneous hyperpigmentation, including minocycline, non-steroidal anti-inflammatory medications, amiodarone, cytotoxic drugs (i.e., busulfan, cyclophosphamide, bleomycin, adriamycin), phenytoin, antipsychotics (i.e., chlorpromazine and related phenothiazines), heavy metals, and antimalarials (chloroquine, hydroxychloroquine) (3). Topical ophthalmic drops have also been associated with ocular tissue pigmentation changes. Most commonly, prostaglandin analogs have been linked to iris hyperpigmentation, periocular hypopigmentation, and eyelash depigmentation (4, 5). Epinephrine eye drops are also associated with hyperpigmentation of the cornea and conjunctiva owing to a light-sensitive oxidation reaction (6). Hyperpigmentation of the sclera is less common, with the tetracycline antibiotic, minocycline, accounting for most reported cases. Minocycline-induced pigment changes are often reported as asymptomatic, bluish color changes that can be isolated to the sclera or can involve cutaneous tissue as well (3).

Hydroxychloroquine (HCQ) is a widely utilized oral medication used to treat a variety of rheumatologic and dermatologic conditions, including systemic lupus erythematosus, dermatomyositis, rheumatoid arthritis, and sarcoidosis, owing to its anti-inflammatory and immunosuppressive effects. However, there are known ocular side effects of this drug, including retinopathy, corneal deposits, and choroid thinning (7). Of note, hyperpigmentation reactions secondary to HCQ are common in the skin, gums, and buccal mucosa, occurring in approximately 29% of patients who were treated with HCQ for at least 6 months (8). These hyperpigmentation reactions have no significant association with the dose or duration of treatment (8). The discoloration can be slowly reversible after cessation of the drug, with improvement possible in the subsequent months; however, there are only a few reports of complete resolution (9).

Although HCQ is known to cause ocular depositions (e.g., corneal verticillata) in 5% of those taking HCQ, there has never been a report of scleral pigmentation changes (7). Herein, we present a unique case report of scleral hyperpigmentation following the use of HCQ.

## Case description

**Figure 1:**
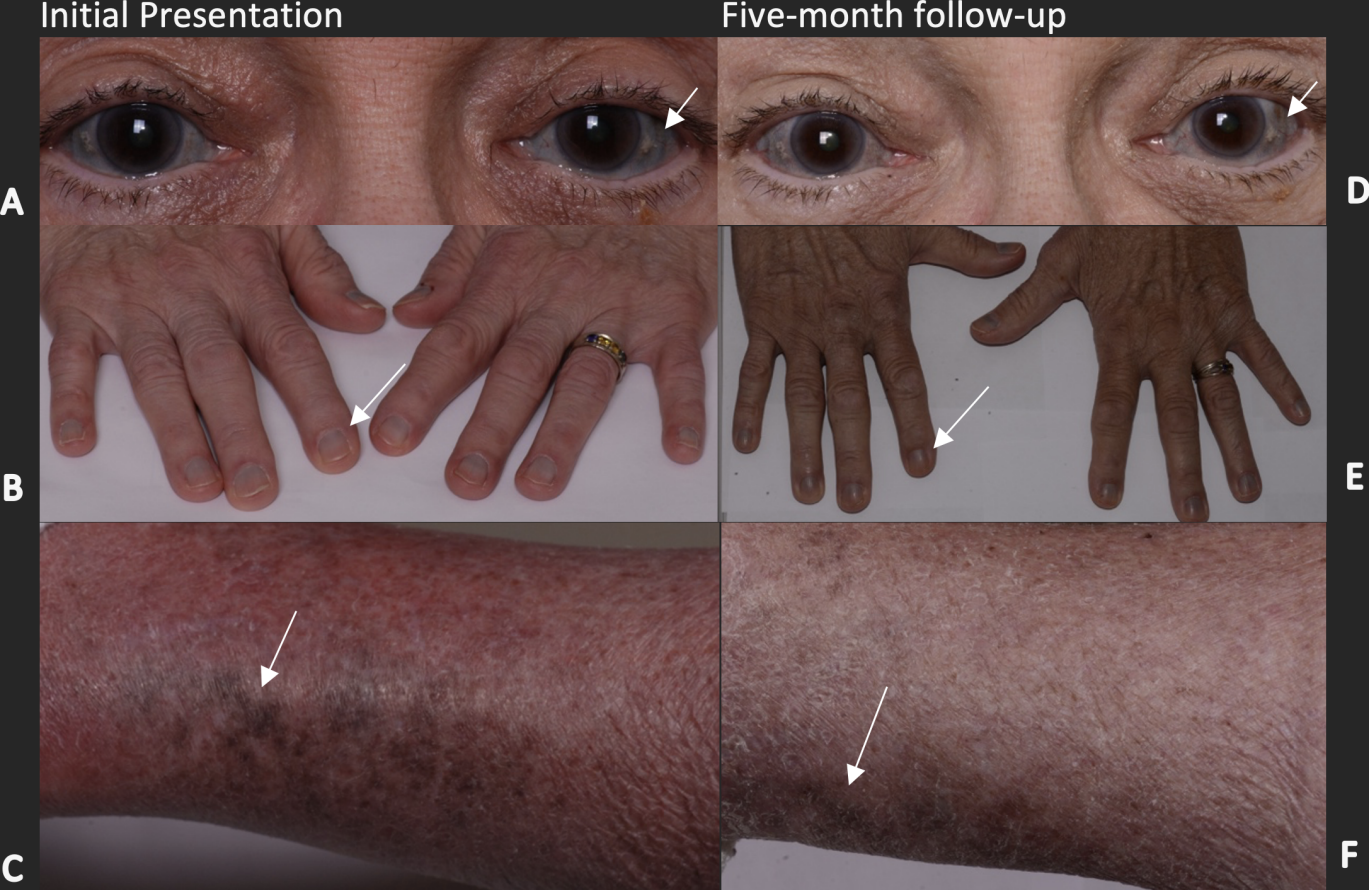
Hyperpigmentation of the right and left sclerae, nail beds, and lower extremities on initial presentation (**A-C**, respectively) and 5 months after cessation of HCQ (**D-F**, respectively). The arrows in **(A, D)** highlight areas of scleral discoloration that are found both medially and temporally. Arrows in **(B, E)** indicate the bluish discoloration of the proximal nailbed, which can be observed in all digits and was slightly improved after 5 months. Arrows in **(C, F)** depict a patchy, bluish-brown discoloration of the lower extremities, which remained similarly discolored but had slightly receded, as depicted by the arrows.

A 75-year-old female presented with a 10-month history of gradual, painless graying discoloration of both sclera, bilateral fingernail beds, and bilateral lower extremities (**Figure 1**). The patient’s medical history was significant for obesity (with a BMI of 39.9 kg/m^2^), Merkel cell carcinoma of the leg, hypertension, atrial fibrillation, and rheumatoid arthritis. She had no history of liver disease or renal disease and at the time of presentation, she had an estimated glomerular filtration rate of 72 ml/minute/1.73 m^2^, which is in the normal range. The patient’s current medications included HCQ, warfarin, metformin, glipizide, metoprolol, simvastatin, losartan, hydrochlorothiazide, and gabapentin. Of note was that she had no history of tamoxifen use. Importantly, she had a remote history of minocycline-induced hyperpigmentation of the skin of her lower extremities after several months of daily minocycline use for rheumatoid arthritis. After discontinuing the minocycline, the pigment changes faded slowly over months and years. This had been in remission for approximately 5 years prior to starting oral HCQ.

In the ophthalmic examination, her best corrected visual acuity was 20/30 both eyes; intraocular pressure, measured by tonometry, was 9 in the right eye and 10 in the left eye; pupils were equal, round, and reactive; and confrontational fields were full in both eyes. The slit lamp was notable for 1+ nuclear sclerosis and pinguecula OU, as well as diffuse graying of the sclera OU without associated scleral thinning or masses (**Figure 1**). The cornea and dilated fundus examination revealed no hyperpigmentation or other abnormalities, and there was no evidence of HCQ-induced retinopathy. No electrophysiologic testing was performed.

## Diagnostic assessment

Given the patient’s history of rheumatoid arthritis, the differential included autoimmune and connective tissue diseases such as scleromalacia perforans and Addison’s disease, which can cause a bronze hyperpigmentation of the sclera. However, there were no systemic symptoms to support Addison’s disease and there was a lack of scleral thinning such that scleromalacia was less likely. Choroidal or ciliary body melanomas were also considered, but there was no evidence of these lesions in the examination. Furthermore, ochronosis was considered, as excess homogentisic acid in alkaptonuria can lead to scleral hyperpigmentation and associated joint pain. However, the patient had no urinary color changes or other symptoms to support this diagnosis.

Given the lack of an organic explanation, we concluded that the oculocutaneous hyperpigmentation was likely drug induced. We considered that this could be a photosensitive exacerbation of more subtle color changes from prior minocycline reactions, but the patient had reported full resolution of symptoms for 5 years after cessation of the drug, and prior eye examinations had all noted normal sclerae. With minocycline unlikely to be responsible, the most likely cause of the hyperpigmentation was determined to be HCQ, as this was the only new medication that she had started within the year prior to presentation, and it has an established role in hyperpigmentation in other tissues of the body. Certain factors have been associated with an increased risk of HCQ retinal toxicity, including obesity and older age, which were both seen in our patient. Higher accumulations of HCQ have been found to accumulate in the ocular tissue of obese patients, which could help explain why this patient was predisposed to scleral discoloration while on the medication (10). Although older age has been historically considered a risk factor for HCQ-related ocular toxicity, more recent studies have older age, in the absence of other comorbidities, to be a poor predictor of ocular toxicity. Meanwhile, other risk factors for ocular HCQ toxicity include renal or hepatic dysfunction and tamoxifen use, but none of these were in our patient’s medical history.

In consultation with rheumatology, the decision was made to discontinue the use of the HCQ to see if this would improve the patient’s findings on follow-up. Given the cosmetic nature of the side effect, and the benefits of immunosuppressant therapy in rheumatoid arthritis, it would have been reasonable to continue with HCQ and monitor for symptom progression. However, as rheumatology recommended alternative immunosuppressants that could achieve the same effect, we determined that cessation of HCQ was appropriate.

**Figure 2:**
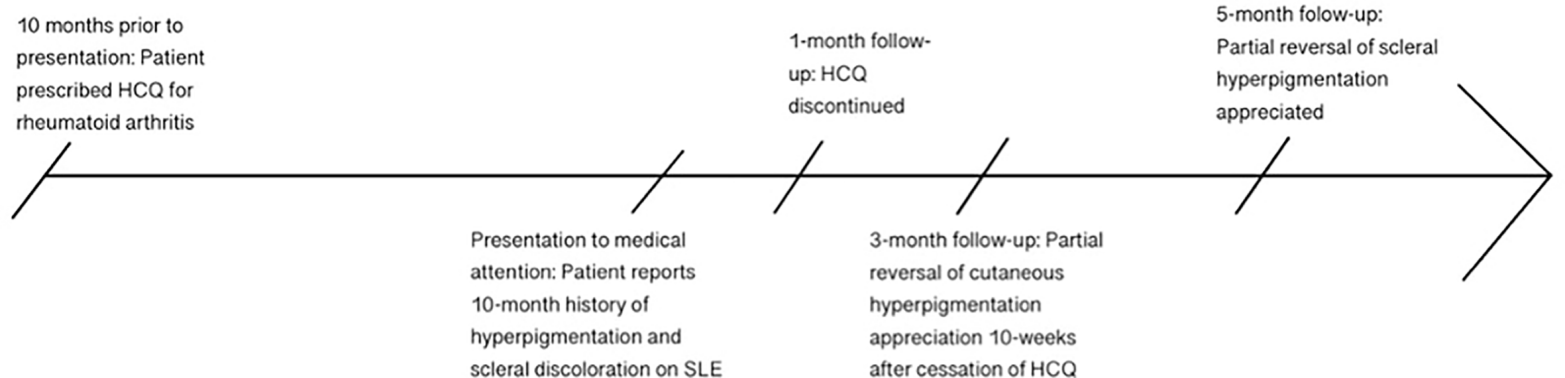
Timeline of patient’s clinical course, from initiation of HCQ to 10 months of gradually worsening hyperpigmentation, followed by discontinuation of HCQ and subsequent slow reversal of discoloration by 5 months after cessation of the drug.

The patient was followed up after 10 weeks, and at that time she reported a gradual improvement in the hyperpigmentation of her nails and legs since the cessation of HCQ; however, pigment changes in the sclera persisted. The patient was followed up again 5 months after the initial presentation and the color changes had improved slightly (**Figures 1**, **2**). This partial reversal of hyperpigmentation on cessation of HCQ further supported it as the causative agent of the systemic hyperpigmentation. However, the lack of substantial improvement in the scleral discoloration suggests a slow-reversal course from months to years.

## Discussion

Hyperpigmentation of the sclera can occur secondary to certain medications and presents as a painless discoloration of the sclera without associated vision changes or systemic symptoms. HCQ is a medication that has been associated with mucocutaneous hyperpigmentation reactions in about 29% of patients (8). However, while other ocular side effects of HCQ are well known, such as retinopathy and corneal verticillate, this is the first reported case, to our knowledge, of hyperpigmentation in the sclera.

HCQ-induced hyperpigmentation in non-ocular sites, such as skin, nails, and mucosa, has no significant association with the dose or duration of HCQ. Despite no significant association, symptom onset occurs after a median of 32 months of treatment and a median cumulative dose of 361 g (8). Of note is that this is less than what the American Academy of Ophthalmology considers “high risk” for HCQ-induced retinopathy: a total cumulative dose of greater than 1,000 g or medication use longer than 5 years (11). Our patient had been taking 400 mg of HCQ daily, for 24 months, with a cumulative dose of 146 g, which is below the median reported above.

The exact mechanism for HCQ-induced hyperpigmentation remains unclear; however, reactions are more likely to occur following microtrauma and in patients on antiplatelet or anticoagulant medications, which is consistent with our patient being on warfarin. These reactions are hypothesized to be due to the increased deposition of hemosiderin and/or melanin in the superficial tissue (12). Our patient had no history of trauma.

Given that HCQ is a commonly used drug for its immunosuppressive and anti-inflammatory properties, it is important to have a clinical suspicion for atypical hyperpigmentation reactions, as this can be cosmetically troubling for patients and potentially lead to decreased treatment adherence. Patients taking long-term HCQ are already recommended to get a regular ophthalmologic follow-up to assess for drug-induced retinopathy, so it is worthwhile to pay careful attention to scleral coloration at these visits as well. As this is the first reported case, it is unknown whether HCQ-induced scleral discoloration could be a predictor of progression to retinopathy and vision loss, but it is possible given the underlying presumption of HCQ accumulation to toxic levels within ocular tissue. That said, HCQ-induced discoloration in non-ocular tissues has been found to occur in a dose-independent fashion in contrast to HCQ-induced retinopathy.

If scleral discoloration is detected, cessation of HCQ can potentially prevent further progression of oculocutaneous changes and even result in a slow, partial reversal of the discoloration.

## Data availability statement

The original contributions presented in the study are included in the article/supplementary material. Further inquiries can be directed to the corresponding author.

## Ethics statement

Written informed consent was obtained from the participant/patient(s) for the publication of this case report

## Author contributions

RW contributed to the conceptual design. CM wrote the first draft of the manuscript. CM and FO contributed background research and analysis. All authors contributed to the article and approved the submitted version.

## References

[1] Gimenez Garcia RM Carrasco Molina S. Drug-induced hyperpigmentation: review and case series. J Am Board Fam Med (2019) 32(4):628–38. doi: 10.3122/jabfm.2019.04.180212 31300585

[2] Krause W. Drug-induced hperpigemntation: a systematic review. JDDG: J der Deutschen Dermatologischen Gesellschaft (2013) 11(7):644–51. doi: 10.1111/ddg.12042 23650908

[3] Phan I Kaiser R Chiu C. A 54-year-old woman with bluish discoloration of her sclera. Digit J Ophthalmol (2010) 16(2):6–8. doi: 10.5693/djo.03.2010.02.002 23362374 PMC3516141

[4] Alm A Grierson I Shields MB. Side effects associated with prostaglandin analog therapy. Surv Ophthalmol (2008) 53 Suppl1:S93–105. doi: 10.1016/j.survophthal.2008.08.004 19038628

[5] Patchinsky A Petitpain N Gillet P Angioi-Duprez K Schmutz JL Bursztejn AC. Dermatological adverse effects of anti-glaucoma eye drops: a review. J Eur Acad Dermatol Venereol (2022) 36(5):661–70. doi: 10.1111/jdv.17928 35032359

[6] Bullock JD. Epinephrine pigmentation. Arch Ophthalmol (1970) 84(4):546–6. doi: 10.1001/archopht.1970.00990040548029

[7] Misra DP Gasparyan AY Zimba O. Benefits and adverse effects of hydroxychloroquine, methotrexate and colchicine: searching for repurposable drug candidates. Rheumatol Int (2020) 40(11):1741–51. doi: 10.1007/s00296-020-04694-2 PMC7467139 32880032

[8] Bahloul E Jallouli M Garbaa S Marzouk S Masmoudi A Turki H. Hydroxychloroquine-induced hyperpigmentation in systemic diseases: prevalence, clinical features and risk factors: a cross-sectional study of 41 cases. Lupus (2017) 26(12):1304–8. doi: 10.1177/0961203317700486 28355984

[9] Coulombe J Boccara O. Hydroxychloroquine-related skin discoloration. CMAJ (2017) 189(5):E212. doi: 10.1503/cmaj.150622 26903361 PMC5289873

[10] Pedrosa T Kupa LVK Pasoto SG Aikawa NE Borba EF Duarte NJ. The influence of obesity on hydroxychloroquine blood levels in lupus nephritis patients. Lupus (2021) 30(4):554–9. doi: 10.1177/0961203320985214 33402039

[11] Iselin KC Marti P Pless M. Hydroxychloroquine-induced retinal toxicity. Klin Monbl Augenheilkd (2016) 233(4):514–6. doi: 10.1055/s-0042-102615 27116524

[12] Puri PK Lountzis NI Tyler W Ferringer T. Hydroxychloroquine-induced hyperpigmentation: the staining pattern. J Cutan Pathol (2008) 35(12):1134–7. doi: 10.1111/j.1600-0560.2008.01004.x 18727667

